# tDCS to premotor cortex changes action verb understanding: Complementary effects of inhibitory and excitatory stimulation

**DOI:** 10.1038/s41598-018-29600-6

**Published:** 2018-07-30

**Authors:** Tom Gijssels, Richard B. Ivry, Daniel Casasanto

**Affiliations:** 1 Department of Psychology, University of Chicago, Chicago, IL 60637 United States of America; 2 Department of Linguistics, Vrije Universiteit Brussel, Brussels, 1050 Belgium; 3 Department of Psychology & Helen Wills Neuroscience Institute, University of California, Berkeley, CA 94720 United States of America; 4 Department of Human Development, Cornell University, Ithaca, NY 14853 United States of America; 5 Department of Psychology, Cornell University, Ithaca, NY 14853 United States of America

## Abstract

Processing the meaning of action language correlates with somatotopic activity in premotor cortex (PMC). A previous neurostimulation study supported a causal contribution of PMC activity to action verb understanding, but the direction of the effect was unexpected: inhibiting PMC made participants respond faster to action verbs. Here we investigated the effects of PMC excitation and inhibition on action verb understanding using tDCS. Right-handed participants received tDCS stimulation with the anodal electrode (presumed to be excitatory) and cathodal electrode (presumed to be inhibitory) placed over left and right PMC, respectively, or with the reverse configuration. After completing the stimulation protocol, participants made lexical decisions on unimanual action verbs (e.g., throw) and abstract verbs (e.g., think). tDCS configuration selectively affected how accurately participants responded to unimanual action verbs. When the anode was positioned over left PMC we observed a relative impairment in performance for right-hand responses (i.e. the hand with which these participants typically perform unimanual actions). By contrast, when the cathode was positioned over left PMC we observed a relative improvement. tDCS configuration did not differentially affect responses to abstract verbs. These complementary effects of excitatory and inhibitory tDCS clarify the functional role of premotor hand areas in understanding action language.

## Introduction

According to theories of embodied cognition, word meaning relies, in part, on neural systems for perceiving and acting^1^. Support for this proposal comes from neuroimaging studies of action language understanding. When people read action verbs like kick, pick, and lick, motor areas show somatotopic activation (i.e. kick, pick and lick preferentially activate leg-, hand-, and mouth-areas, respectively^2^; see Pulvermüller, 2005, for review^3^).

What is the motor system contributing to action verb understanding? On one view, motor simulations recapitulate previous action experiences by re-activating some of the neural circuits used to perform those actions^1,3^. According to this re-enactment account, Hebbian learning creates connections between neural circuits involved in action execution (most critically primary motor cortex; M1), and neural representations of the action verb word forms^3^. On an alternative view, motor simulations may partially prepare the motor system for future actions – thus, simulations are “pre-enactments” rather than reenactments^4–6^. If the pre-enactment view is correct, motor simulations should be implemented primarily in neural systems that support action planning, instead of action execution. fMRI data support this proposal: processing action verbs correlates mainly with activity in motor planning areas (e.g., premotor cortex; PMC), rather than activity in motor execution areas (e.g., primary motor cortex^4,7,8^; but see^3^).

To date, only one study tested the functional role of PMC in action verb understanding. Willems *et al*.^9^ used continuous theta-burst stimulation (cTBS) to perturb activity in either left or right PMC areas involved in planning right- and left-hand actions, respectively. Right-handed participants then made lexical decisions on unimanual and nonmanual action verbs. They responded faster to unimanual action verbs after cTBS to left PMC than after cTBS to right PMC. By contrast, cTBS to left vs. right PMC did not differentially affect responses to nonmanual action verbs^9^. fMRI evidence suggests that these effects were likely driven by cTBS to dominant hand areas in left PMC: When right-handers processed unimanual action verbs in the scanner, they showed BOLD modulation in right-hand areas in left PMC, but not in left-hand areas in right PMC^10^.

Yet, the direction of Willems *et al*.’s^9^ reaction time pattern was unexpected. cTBS has been shown to suppress neural excitability^11^. On the simplest prediction, left PMC stimulation might be expected to impair, instead of improve, performance. Therefore, Willems *et al*.’s data may be an instance of “paradoxical functional facilitation,”^12^: Patients with brain lesions to inhibitory circuits sometimes show enhanced behavioral performance, relative to controls^12,13^. Likewise, cTBS to left PMC might have facilitated action verb processing by modulating inhibitory PMC circuits^14–17^. On one possibility, inhibition may prevent people from overtly performing the actions named by verbs, rather than covertly simulating them. For instance, when people read a verb like “kick”, inhibition may be necessary to stop people from performing an actual kicking movement (except, of course, if this action is contextually appropriate)^9^. This post-hoc explanation generates testable predictions regarding the effects of inhibitory vs. excitatory stimulation of left PMC: Whereas inhibitory stimulation to left PMC should improve performance on action verbs, excitatory stimulation of the same region should impair performance.

Here we used transcranial Direct Current Stimulation (tDCS) to test for complementary effects of excitatory and inhibitory left PMC stimulation on action verb understanding. TDCS passes a weak direct current between two scalp electrodes, increasing neural excitability under the anodal electrode and decreasing excitability under the cathodal electrode^18,19^. Right-handers received tDCS over hand areas in left and right PMC, with the left-right configuration of the anode and cathode counterbalanced across participants. Following stimulation, participants performed a lexical decision task involving unimanual and abstract verbs, responding with their left and right hands.

We expected that inhibitory, cathodal stimulation over left PMC hand area would cause an improvement in processing unimanual action verbs, as found by Willems *et al*. By contrast, excitatory, anodal stimulation to the same area was expected to cause an impairment in processing unimanual action verbs, thereby complementing the pattern found by Willems *et al*.

## Results

### Accuracy

**Figure 1:**
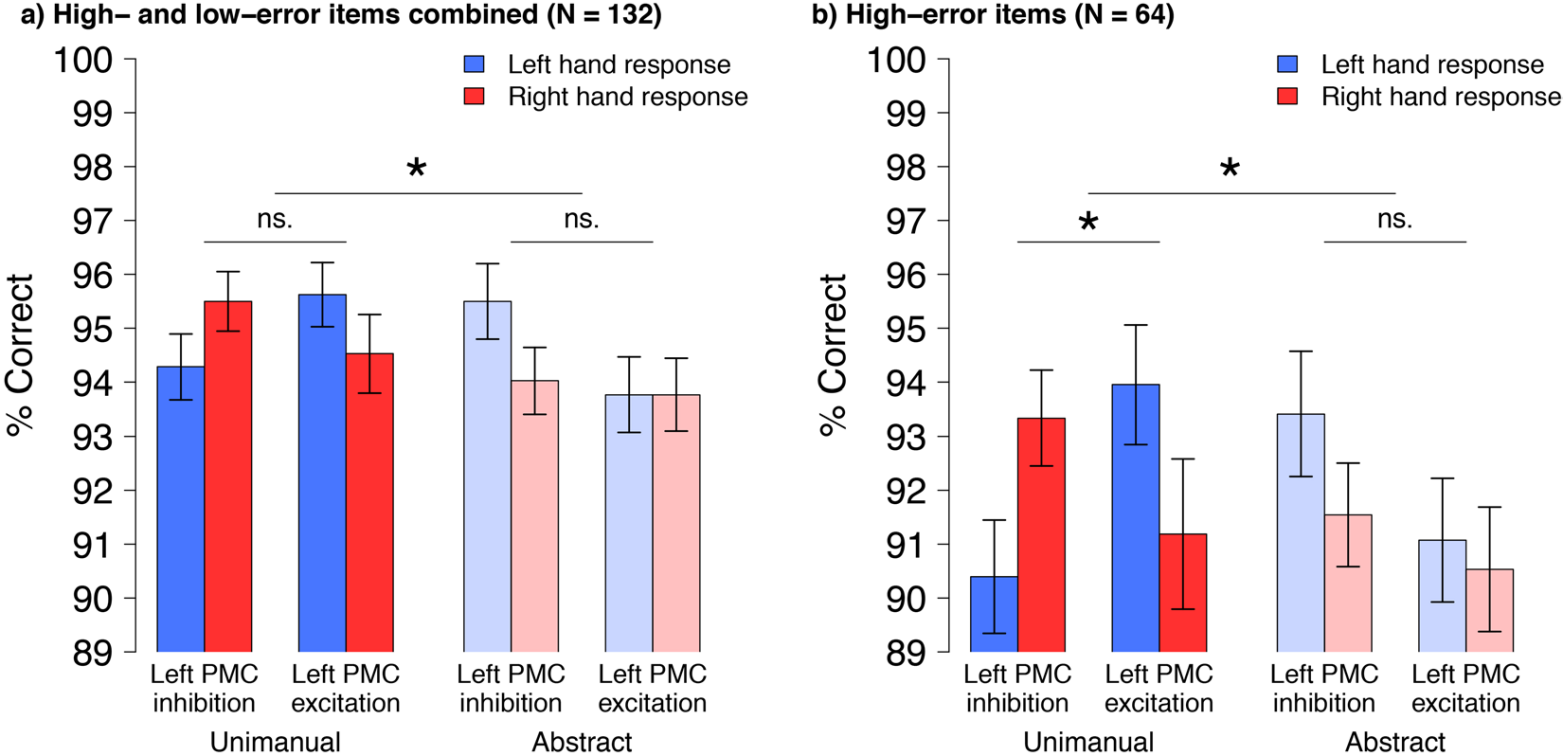
Mean accuracy on the lexical decision task for (**a**) all items and (**b**) items with a high error rate. Left PMC Inhibition = cathode left PMC, anode right PMC; Left PMC Excitation = anode left PMC, cathode right PMC. Unimanual and Abstract refer to the Verb type of the stimuli. Error bars reflect within- subject SEM. *Indicates p < 0.05.

The polarity of tDCS to left PMC differentially affected the accuracy of participants’ responses to unimanual vs. abstract verbs, as indicated by a significant 3-way interaction of tDCS polarity × Verb type × Response hand (β = −0.82, SE = 0.40, z = −2.07, p = 0.04; Fig. 1a). In addition to the predicted 3-way interaction, we also found the predicted qualitative pattern of results for the constituent 2-way interactions. This qualitative pattern showed that inhibitory left PMC stimulation tended to cause a relative improvement in performance for right-hand responses, whereas excitatory left PMC stimulation tended to cause a relative impairment; by contrast, tDCS polarity had no systematic effect on accuracy for abstract verbs. This pattern suggests that the predicted relationship between tDCS polarity and response hand was selective for unimanual verbs, yet the 2-way interaction needed to confirm this inference showed the predicted pattern, qualitatively, but was not statistically significant (β = −0.32, SE = 0.32, z = −0.98, p = 0.33).

One possible reason why the predicted 2-way interaction in the unimanual verb condition did not reach significance is that accuracy approached 100%, in all conditions: a ceiling effect. To determine whether the predicted effects were masked by this ceiling effect, we performed a second analysis on a subset of items that were not near ceiling. We calculated the number of errors for each verb (Range = 0–16 errors, Median = 3) and then performed a median split to identify items that led to more errors (High-error items: n = 31 unimanual verbs, n = 33 abstract verbs; Low-error items: n = 35 unimanual verbs, n = 33 abstract verbs).

The accuracy analysis of high-error items again showed the predicted 3-way interaction of tDCS polarity ×  Verb type × Response hand (β = −1.07, SE = 0.47, z = −2.29, p = 0.02; Fig. 1b). As expected, this 3-way interaction was driven selectively by responses to unimanual verbs, as shown by a significant 2-way interaction of tDCS polarity × Response hand (β = 0.80, SE = 0.35, z = −2.32, p = 0.02; Fig. 1b). After inhibitory stimulation to left PMC, participants tended to respond more accurately to unimanual verbs with their right hand than with their left hand (β = −0.41, SE = 0.26, z = −1.58, p = 0.11; Fig. 1b). After excitatory stimulation to left PMC, we found a trend in the opposite direction: participants tended to respond less accurately with their right hand than with their left hand (β = 0.62, SE = 0.37, z = 1.67, p = 0.10; Fig. 1b). As expected, there was no statistically significant 2-way interaction of tDCS polarity and Response hand for abstract verbs (β = 0.20, SE = 0.36, z = 0.56, p = 0.58; Fig. 1b).

Although not of interest, for completeness we report that analysis of the low-error (near-ceiling) items showed no evidence for a 3-way interaction of tDCS polarity x Verb type x Response hand (β = −0.18, SE = 0.74, z = −0.25, p = 0.80) nor for either of the 2-way interactions of tDCS polarity x Response hand (unimanual verbs: β = 0.08, SE = 0.68, z = 0.11, p = 0.91; abstract verbs: β = 0.43, SE = 0.65, z = 0.66, p = 0.51).

### Reaction Times

RTs were defined as the latency from stimulus onset to release of the “home” button. There were no statistically significant effects for the 3-way interaction of tDCS polarity × Verb type × Response hand (Wald χ^2^ (1) = 0.68, p = 0.41, see Supplementary Fig. S1), nor for the constituent 2-way interactions of tDCS polarity × Response hand for either verb type (unimanual: Wald χ^2^ (1) = 0.10, p = 0.75; abstract: Wald χ^2^ (1) = 0.31, p = 0.58). As with the accuracy data, we analyzed the release RTs separately for the high- and low-error items. There were no statistically significant 3-way or 2-way interactions in either model (all Wald χ^2^s < 1.94; all ps > 0.15). The lack of any RT effect argues against a speed-accuracy trade-off as an explanation for our observed accuracy effects.

## Discussion

This study tested whether motor circuits involved in action preparation play a causal role in action verb understanding. To this end, we used two tDCS configurations to alter the state of excitability in hand areas of premotor regions. For one configuration, the anodal electrode was positioned over the left PMC and the cathodal electrode over the right PMC, a configuration assumed to enhance excitability in left PMC. For the other configuration, the electrodes were reversed to reduce excitability (or promote an inhibitory state) in left PMC. Inhibitory and excitatory tDCS to left PMC differentially affected the accuracy of responses to unimanual action verbs, but not to abstract verbs. After left PMC inhibition, right-handers tended to make fewer errors to unimanual verbs with their right hand than with their left hand. After left PMC excitation, right-handers tended to make more errors to unimanual verbs with their right hand than with their left hand. By contrast, the configuration of tDCS did not differentially influence how accurately participants responded to abstract verbs. These results demonstrate complementary effects of exciting and inhibiting left PMC activity on action verb processing.

Neurostimulation to motor areas has been shown to benefit action language processing, but previous studies have provided “no evidence yet that sensory-motor cortex stimulation disrupts [i.e., impairs] semantic processing,”^20^ (p. 6; see also^21,22^). Here, depending on the configuration of tDCS and on the response hand, we find both a relative improvement and a relative impairment in accuracy for lexical decisions, using a task known to produce semantic priming effects^23^.If we assume that tDCS-induced inhibition is similar to disruption caused by cTBS, then the current results are similar to those reported by Willems *et al*.^9^: Both conditions produced “paradoxical facilitation” of lexical decision after inhibition or disruption of left PMC hand areas. Conversely, we showed “paradoxical impairment” after excitation of the same area. One explanation for the complementary effects of excitatory and inhibitory neurostimulation builds on the idea that there is competition between different possible simulations of the same action. For instance, given the verb “throw,” people could simulate either an overhand or an underhand throw. Perhaps both of these simulations are partially activated initially, but since these actions cannot be performed simultaneously, the developing simulations compete and one “wins.” Left-inhibitory stimulation could facilitate performance by reducing activation of competing simulations, whereas left-excitatory stimulation may impair performance by increasing this competition (see^9,14–17^).

An alternative explanation of these complementary effects of tDCS is based on the idea that inhibition of modality-specific areas may minimize the conflict between simulations and task-relevant perceptual or motor processes. Analogous to our results in the motor modality, Landau and colleagues^24^ (see also^25^) found that after people process language about faces, they show impaired (rather than improved) behavioral and neural responses to pictures of faces. In both Landau *et al*.’s study and our own, words may have cued activity in the relevant perceptual-motor systems, but this activity needed to be inhibited because the task did not call for the corresponding percepts or actions (e.g., given the verb “throw,” our participants were not expected to throw, but rather to press a button). On this account, PMC inhibition may help ensure that simulations do not result in the overt execution of the action named by a verb. Left-inhibitory stimulation may have improved behavioral performance by increasing inhibition of situationally inappropriate motor plans. Left-excitatory stimulation may have impaired performance by boosting activation of all potentially relevant motor plans (i.e., both the verb-cued simulations and preparations for the manual response), thus increasing competition among these representations. Further experiments are needed to test these potential explanations, and to determine the mechanisms underlying the paradoxical improvement and impairment effects we observed.

The current data also provide evidence that motor system activity affects how well people process action verbs. Previous neurostimulation results showed that changing activity in the motor system affects the speed with which people process action verbs^9,26^. Yet, as Willems and Casasanto^27^ point out, these data do not address how motor activity changes the quality with which people understand these verbs. Here we show that stimulation of hand areas affects how accurately participants process manual action verbs. In combination with previous findings, these results suggest that motor simulations contribute to both how fast and how well people construct the meaning of action language.

Why did we observe the predicted pattern in the accuracy data but not in the RT data? Since we did not speed participants (e.g., with a trial timeout), it may not be surprising that we observed the predicted interactions in the accuracy data, alone. Furthermore, the absence of an RT effect allows us to rule out the presence of a speed-accuracy trade-off. In general, it is often unclear a priori whether studies investigating motor-system contributions to action language understanding will show the predicted effect in RTs, accuracy, or both (see^22^). Across our cTBS and tDCS studies, we find the predicted effects in both reaction times and accuracy. Since finding the predicted effect in either accuracy or RT could be interpreted as support for our experimental hypothesis, a reasonable (though non-standard) precaution to take would be to double our p-values (i.e. Bonferroni correction). Even if we do so, the critical 3-way and 2-way interactions remain significant in our high-error data set.

We observed the predicted 3-way interaction of tDCS polarity, verb type, and response hand in the full data set, but the predicted 2-way interaction of tDCS polarity × response hand was not significant, presumably due to a ceiling effect. Overall, these results should be interpreted with some caution given that we had to perform an unplanned median split in order to observe the predicted 2-way interaction of tDCS polarity by response hand. Our confidence in the data is increased, however, by the facts that (a) the critical 3-way interaction was significant in all analyses, (b) inhibitory left PMC stimulation produced a close conceptual replication of our previous cTBS study, and (c) excitatory left PMC stimulation produced the predicted mirror image of these results, providing further convergent evidence that left PMC activity plays a functional role in processing unimanual action verbs.

In this study, we targeted PMC, rather than primary motor cortex (M1), because we hypothesize that PMC is associated with implicit simulations underlying language processing, whereas M1 is involved in overt action execution and explicit motor imagery: a hypothesis supported by previous fMRI data^4,10^. Studies reporting a functional role of M1 in action language processing often rely on tasks that encourage participants to use imagery. In one study^28^, participants received tDCS to M1 hand areas while they made concreteness judgments on hand- and leg-action verbs, a task which requires deep semantic processing and is conducive to imagery. The results showed that cathodal tDCS made participants faster to judge the concreteness of action verbs (compatible with the current results) but also showed that this RT improvement was restricted to those participants who were doing the deepest semantic processing, and who were therefore the most likely to rely on explicit imagery^28^. Previous studies that stimulated M1 to test its functional role in action language processing have produced somewhat inconsistent findings^26,29–33^. Whether or not M1 stimulation affects action verb processing may depend, in part, on whether the tasks encourage participants to explicitly imagine motor actions (which appears to depend on M1 as well as PMC), or only to implicitly simulate them (which appears to depend primarily on PMC, rather than M1^4,10^).

The pattern of results described above is compatible with the proposal that implicit simulations during lexical decision constitute partial preparation for the actions named by the verbs, and should therefore rely more on action planning areas (PMC) than on areas directly involved in action execution (M1). Yet, the low spatial resolution of tDCS leaves open the possibility that the current spread to nearby cortical regions, including ipsilateral hand areas in M1. Nonetheless, several factors make it likely that our results are driven by stimulation of PMC rather than M1. First, the tDCS montage was specifically set up to target PMC: We determined the appropriate electrode positions for targeting PMC hand-areas using MNI coordinates from previous fMRI work^10^ and using MNI-to-electrode positioning conversion tables^34^. As the current density is the highest under the focal point of stimulation^35^, PMC hand areas were receiving the most focal stimulation. Second, the literature on action language processing provides both theoretical and empirical motivation for the idea that shallow language processing tasks (like lexical decision) rely on PMC and not M1. Still, we cannot definitively rule out the possibility that M1 was also affected by our tDCS stimulation. If M1 stimulation contributed to the present results this would call for a modification of our arguments about the neural bases of explicit imagery vs. implicit simulation (since M1 is implicated more strongly in the former than the latter), but the other theoretical inferences we are making from these data would remain unchanged.

Finally, although our tDCS montage was designed to target left PMC, we also simultaneously stimulated right PMC. Could the current pattern of results be explained by inhibitory or excitatory stimulation to right PMC, alone? Based on the results from previous studies, the answer appears to be: No. When right-handed participants process unimanual action verbs, they primarily rely on left PMC hand circuits, but not on right PMC hand circuits. This pattern is now supported by two fMRI studies^4,10^ and one cTBS study^9^. This lateralized pattern of activity is consistent with the proposal that right-handers understand language about unimanual actions by relying on the same circuits they typically use to perform those actions: left PMC circuits that control the right hand. Nonetheless, even though stimulation of right PMC alone cannot fully account for the present results, changes in right PMC activity could have strengthened the observed pattern. Because left and right PMC are connected through transcallosal pathways, stimulation of right PMC could have affected activity in left PMC through interhemispheric inhibition^36,37^, which may have increased the inhibitory effects of ongoing cathodal stimulation to left PMC. Yet, even if interhemispheric inhibition played a role in strengthening our results, this mechanism does not change the inference that left PMC plays a functional role in understanding manual action language, and that stimulating left PMC produces the observed paradoxical effects.

To conclude, these results point to a functional relationship between neural systems for planning hand actions and for understanding language about those actions. tDCS to PMC affected how accurately people processed action language: A configuration that induced inhibition of left PMC caused a relative improvement in performance (consistent with our previous cTBS results), whereas a configuration that induced excitation of left PMC caused a relative impairment. These complementary effects of excitatory and inhibitory tDCS were specific to unimanual action verbs, and depended critically on the hand that participants used to respond. Previous neurostimulation results have shown that modulating PMC activity can influence how fast people respond to action verbs. The present results show that modulating PMC activity in the hemisphere that controls the dominant hand can also affect how well people process verbs that name dominant-hand actions, strengthening the evidence that motor simulations contribute to the meanings of action words.

## Methods

### Participants

73 participants from the University of Chicago community took part in the experiment. Data from 1 participant who did not follow the task instructions were replaced, and data from 1 other participant were lost due to a script error. The remaining 71 participants (33 females, 38 males) were monolingual native English speakers and were right-handed as established by the Edinburgh Handedness Inventory (EHI: M = 78; range = 47–100^38^). Participants were healthy adults who did not report being pregnant, having sustained a stroke or brain injury, being on psychoactive medication, or having any electronic implants. All participants provided informed consent and received course credit or $30 for their participation. All procedures were in accordance with the guidelines approved by the Institutional Review Board of the University of Chicago.

### Materials and Procedure

#### Stimuli

198 verbs were used in this experiment: 66 unimanual verbs (e.g. to write), 66 abstract verbs (e.g. to tempt), and 66 phonotactically legal nonce words (e.g. to frinckle). Unimanual action verbs were selected based on the results of a pantomime rating study. In the pantomime study, 10 participants read each of the unimanual action verbs used by Willems *et al*.^9^ and acted out the actions described by them. All participants had a strong hand preference, as established by the Edinburgh Handedness Inventory (8 strong right-handers: EHI: M = 92; range = 70–100; 2 strong left-handers: EHI: M = −100, range = −100). For each pantomime, we coded which hand participants used to perform the action. Our final stimulus set only included those verbs that elicited primarily dominant hand responses (lower bound cut-off set at 60% dominant hand responses; total N of final stimulus set = 66; proportion dominant hand responses: M = 90%; range = 60–100%).

Although we used a within-item design, all three verb types were matched in word length (unimanual vs. abstract: t(130) = 1.48, p = 0.14; unimanual vs. nonce: t(130) = −0.74, p = 0.46; abstract vs. nonce: t(130) = −0.74, p = 0.46). Unimanual and abstract verbs were matched in word frequency (t(96) = 0.20; p = 0.85^39^).

#### Transcranial Direct Current Stimulation

tDCS was performed using a battery-powered Soterix Medical 1 × 1 (Soterix Medical, New York) with two 5 × 7 cm saline-soaked sponges covering the electrodes. Each participant received 20 minutes of stimulation at 2 mA, which was slowly ramped up from 0 mA at stimulation onset, and ramped down to 0 mA at stimulation offset. Both ramping up and ramping down happened over the course of 20 seconds. The electrodes were placed over premotor hand areas, at FC3 and FC4 in the 10–20 electrode system^18,34^. We selected FC3 and FC4 because these electrodes provided the closest overlap with the MNI coordinates of PMC hand areas (MNI coordinates were based on fMRI data from^10^, MNI to electrode mapping was based on the conversion table provided by Koessler *et al*.^34^).

In the left PMC inhibition condition (N = 35), the cathode was placed at FC3 and the anode at FC4, inhibiting left PMC and simultaneously exciting right PMC. In the left PMC excitation condition (N = 36) this placement was reversed, with the anode placed at FC3 and the cathode at FC4, exciting left PMC and inhibiting right PMC. All participants tolerated stimulation; one participant expressed discomfort. During stimulation, participants did not perform any task and were asked to refrain from moving. We selected this approach, first, because performing motor actions during tDCS can change the effects of tDCS, and second, because the effects of tDCS persist up to an hour after stimulation (see^18^).

#### Behavioral Procedure

After receiving tDCS participants performed a lexical decision task. Verbs appeared one at a time in the center of a computer screen. Participants indicated whether each stimulus was an existing English word by pressing a button corresponding to “yes” or “no” with their left or right index finger. The response mappings for each button were presented below the verb, on the left or right side of the screen. For each verb type, the “yes” response was mapped to the right button for half of the stimuli and to the left button for the other half (mapping counterbalanced across participants). The stimuli appeared in a random order, and the placement of the response labels varied unpredictably from one trial to the next.

**Figure 2:**
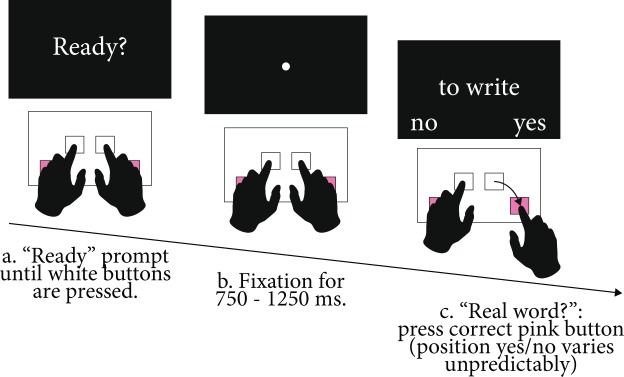
Typical lexical decision trial. (**a**) “Ready?” prompt until participants pressed and held down the two white buttons. (**b**) Fixation for a random duration between 750–1250 ms. Participants kept holding down the white buttons. (**c**) The stimulus and response labels appeared, participants formulated their response and pressed the pink button corresponding to the correct response.

Every trial had the following structure (see Fig. 2). First, participants saw a “Ready?” sign prompting them to push and hold down the two white “home” buttons with their left and right index finger (mapped to the ‘d’ and ‘k’ keys). Once the buttons were held down, a fixation cross appeared for a duration randomly selected between 750 and 1250 ms. Then, the stimulus and response prompts appeared. As soon as the participant had decided the correct response, they released the home button held down by the response hand and used the same hand to push the correct pink response button, after which a new trial started. Response buttons were mapped to the ‘z’ and ‘period’ keys. If participants released either of the home buttons before the stimulus was presented, the trial was restarted. If participants released or pressed the wrong buttons in response to the stimulus, they received feedback and the response was classified as incorrect.

#### Data analysis

The accuracy data and RTs for target trials were analyzed with mixed effects models using the ‘lme4’ package^40^ for R^41^. The independent variables for both models consisted of three two-level fixed effects: tDCS polarity (left inhibitory vs. left excitatory); verb type (unimanual vs. abstract), and response hand for the required response (left vs. right). We included random effects for participant and word, and used the maximal random effects structure^42^. Nonce trials were excluded before the analyses. Accuracy data were analyzed using a general linear model with a binomial linking function. The dependent variable for this model was whether the response for each trial was correct or incorrect. For the RT model, we discarded all the incorrect trials and log-transformed RTs to reduce skew in the residuals.

Both the accuracy and RT models used the following formula: DV (Accuracy/RT) ~ tDCS Polarity × Verb Type × Response Hand + (1 + Verb Type × Response Hand | Participant) + (1 + tDCS Polarity x Response Hand | Word).

Finally, to test the constituent interactions of the 3-way interactions, we used separate models to test for lower-order interactions for each verb type (e.g. the 2-way interaction of tDCS Polarity × Response Hand for unimanual action verbs and abstract verbs). These models had the exact same fixed and random effect structure as the full, higher-order model, except that they did not include the factor Verb Type. Outputs for each model are included in Supplementary Tables 1–9.

### Data availability statement

The datasets generated during and analyzed during the current study are available in the OSF repository, at http://osf.io/rxfye.

## Electronic supplementary material

Supplementary Materials

## References

[1] Barsalou LW (1999). Perceptual symbol systems. Behav. Brain Sci..

[2] Hauk O, Johnsrude I, Pulvermüller F (2004). Somatotopic representation of action words in human motor and premotor cortex. Neuron.

[3] Pulvermüller F (2005). Brain mechanisms linking language and action. Nat. Rev. Neurosci..

[4] Willems RM, Toni I, Hagoort P, Casasanto D (2010). Neural dissociations between action verb understanding and motor imagery. J. Cogn. Neurosci..

[5] Zwaan, R. A. & Kaschak, M. P. Language in the brain, body, and world in *The Cambridge handbook of situated cognition* (eds Robbins, P. & Aydede, M.) 368–381 (Cambridge University Press, New York, 2008).

[6] Gijssels, T. & Casasanto, D. tDCS to premotor cortex changes action verb understanding: Complementary effects of inhibitory and excitatory stimulation in Proc. of the 38th Ann. Conf. of the Cog. Sci. Soc. (eds. Papafragou, A., Grodner, D., Mirman, D., & Trueswell, J.C.) 2717–2722 (2016). 10.1038/s41598-018-29600-6 PMC6065381 30061670

[7] Aziz-Zadeh L, Wilson SM, Rizzolatti G, Iacoboni M (2006). Congruent embodied representations for visually presented actions and linguistic phrases describing actions. Curr. Biol..

[8] Tettamanti M (2005). Listening to action-related sentences activates fronto-parietal motor circuits. J. Cogn. Neurosci..

[9] Willems RM, Labruna L, D’Esposito M, Ivry R, Casasanto D (2011). A functional role for the motor system in language understanding evidence from theta-burst transcranial magnetic stimulation. Psychol. Sci..

[10] Willems RM, Hagoort P, Casasanto D (2010). Body-specific representations of action verbs neural evidence from right-and left-handers. Psychol. Sci..

[11] Huang Y-Z, Edwards MJ, Rounis E, Bhatia KP, Rothwell JC (2005). Theta burst stimulation of the human motor cortex. Neuron.

[12] Kapur N (1996). Paradoxical functional facilitation in brain-behaviour research. A critical review. Brain.

[13] Papeo L, Pascual-Leone A, Caramazza A (2013). Disrupting the brain to validate hypotheses on the neurobiology of language. Front. Hum. Neurosci..

[14] Prut Y, Fetz EE (1999). Primate spinal interneurons show pre-movement instructed delay activity. Nat..

[15] Sawaguchi T, Yamane I, Kubota K (1996). Application of the GABA antagonist bicuculline to the premotor cortex reduces the ability to withhold reaching movements by well-trained monkeys in visually guided reaching task. J. Neurophysiol..

[16] Kroeger J (2010). Charting the excitability of premotor to motor connections while withholding or initiating a selected movement. Eur. J. Neurosci..

[17] Duque J, Labruna L, Verset S, Olivier E, Ivry RB (2012). Dissociating the role of prefrontal and premotor cortices in controlling inhibitory mechanisms during motor preparation. J. Neurosci..

[18] Nitsche MA (2008). Transcranial direct current stimulation: state of the art 2008. Brain Stimul..

[19] Jacobson L, Koslowsky M, Lavidor M (2012). tDCS polarity effects in motor and cognitive domains: a meta-analytical review. Exp. Brain Res..

[20] Hauk O, Tschentscher N (2013). The body of evidence: what can neuroscience tell us about embodied semantics?. Front. Psychol..

[21] Mahon BZ, Caramazza A (2005). The orchestration of the sensory-motor systems: Clues from neuropsychology. Cogn. Neuropsychol..

[22] Mahon BZ, Caramazza A (2008). A critical look at the embodied cognition hypothesis and a new proposal for grounding conceptual content. J. physiology-Paris.

[23] Neely JH, Keefe DE, Ross KL (1989). Semantic priming in the lexical decision task: roles of prospective prime-generated expectancies and retrospective semantic matching. JEP: LMC.

[24] Landau AN, Aziz-Zadeh L, Ivry RB (2010). The influence of language on perception: listening to sentences about faces affects the perception of faces. J. Neurosci..

[25] Aziz-Zadeh L (2008). Modulation of the FFA and PPA by language related to faces and places. Soc. Neuroscience.

[26] Pulvermüller F, Hauk O, Nikulin VV, Ilmoniemi RJ (2005). Functional links between motor and language systems. Eur. J. Neurosci..

[27] Willems RM, Casasanto D (2011). Flexibility in embodied language understanding. Front. Psychol..

[28] Niccolai V, Klepp A, Indefrey P, Schnitzler A, Biermann-Ruben K (2017). Semantic discrimination impacts tDCS modulation of verb processing. Sci. Reports.

[29] Liuzzi G (2010). The involvement of the left motor cortex in learning of a novel action word lexicon. Curr. Biol..

[30] Vicario CM, Rumiati RI (2012). tDCS of the primary motor cortex improves the detection of semantic dissonance. Neurosci. letters.

[31] Papeo L, Vallesi A, Isaja A, Rumiati RI (2009). Effects of TMS on different stages of motor and non-motor verb processing in the primary motor cortex. PLoS One.

[32] Tomasino B, Fink GR, Sparing R, Dafotakis M, Weiss PH (2008). Action verbs and the primary motor cortex: a  comparative TMS study of silent reading, frequency judgments, and motor imagery. Neuropsychol..

[33] Lo Gerfo E (2008). The influence of rTMS over prefrontal and motor areas in a morphological task: grammatical vs. semantic effects. Neuropsychol..

[34] Koessler L (2009). Automated cortical projection of EEG sensors: anatomical correlation via the international 10–10 system. Neuroimage.

[35] Wagner T (2007). Transcranial direct current stimulation: a computer-based human model study. Neuroimage.

[36] Daskalakis ZJ, Christensen BK, Fitzgerald PB, Roshan L, Chen R (2002). The mechanisms of interhemispheric inhibition in the human motor cortex. The J. physiology.

[37] Bestmann S (2007). Dorsal premotor cortex exerts state-dependent causal influences on activity in contralateral primary motor and dorsal premotor cortex. Cereb. Cortex.

[38] Oldfield RC (1971). The assessment and analysis of handedness: the Edinburgh inventory. Neuropsychol..

[39] Coltheart M (1981). The MRC psycholinguistic database. The Q. J. Exp. Psychol..

[40] Bates, D., Mächler, M., Bolker, B. & Walker, S. Fitting linear mixed-effects models usinglme4. *arXiv preprint arXiv:1406.5823* (2014).

[41] R Core Team. *R: A Language and Environment for Statistical Computing*. R Foundation for Statistical Computing, Vienna, Austria http://www.R-project.org/ (2014).

[42] Barr DJ, Levy R, Scheepers C, Tily HJ (2013). Random effects structure for confirmatory hypothesis testing: Keep it maximal. JML.

