# Supplementary material for: tDCS to premotor cortex changes action verb understanding: Complementary effects of inhibitory and excitatory stimulation
Source: Sci Rep. 2018 Jul 30;8:11452. doi: 10.1038/s41598-018-29600-6 (PMC6065381; doi:10.1038/s41598-018-29600-6)
Supplement: Supplementary file 1 — Supplementary Materials [file 41598_2018_29600_MOESM1_ESM.pdf]

Supplementary Materials.

tDCS to premotor cortex changes action verb understanding: Complementary effects of inhibitory and excitatory stimulation

Tom Gijssels[1,2], Richard B. Ivry[3], and Daniel Casasanto[1]

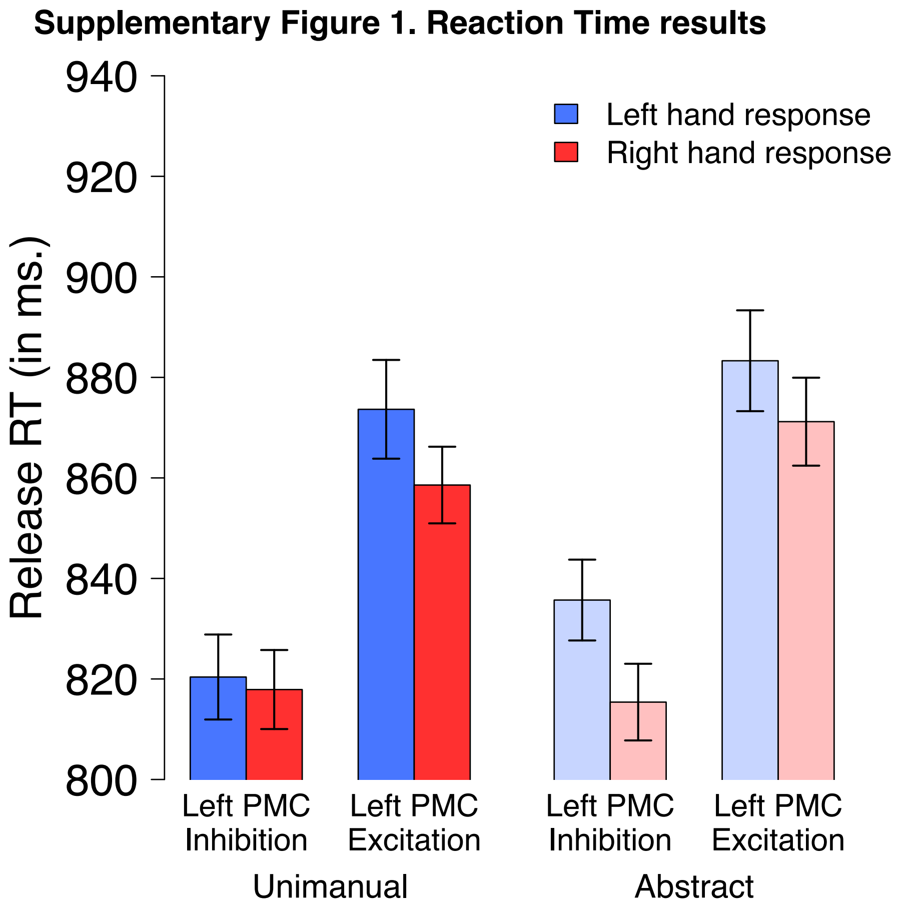

Supplementary Figure 1. Mean release reaction time on the lexical decision task for all items. Left PMC Inhibition = cathode left PMC, anode right PMC; Left PMC Excitation = anode left PMC, cathode right PMC. Unimanual and Abstract refer to the Verb type of the stimuli.

Supplementary Table 1. Full model: High- and low-error items combined

	Estimate	SE	z-value	p
Intercept	-3.13	.001	-5,948.231	<0
Anode Position	-.105	.157	.157	.504
Verb Type	-.204	.14	-1.461	.144
Resp. Hand	.073	.131	.556	.578
Anode Position x Verb Type	.26	.205	1.269	.205
Anode Position x Resp. Hand	-.003	.153	-.018	.986

Verb Type x Resp. Hand	-.163	.211	.771	.44
Anode Position x Verb Type x Resp. Hand	-.818	.396	-2.065	.039

Supplementary Table 2. Submodel for unimanual verbs: High- and low-error items combined

	Estimate	SE	z-value	p
Intercept	-3.195	.127	-25.188	0
Anode Position	.07	.213	.327	.743
Resp. Hand	-.029	.191	-.15	.881
Anode Position x Resp. Hand	-.317	.323	.983	.326

Supplementary Table 3. Submodel for abstract verbs: High- and low-error items combined

	Estimate	SE	z-value	p
Intercept	-3.018	.085	-35.444	0
Anode Position	-.266	.182	-1.466	.143
Resp. Hand	.173	.179	.965	.334
Anode Position x Resp. Hand	.333	.314	1.06	.289

Supplementary Table 4. Full model: High-error items only

	Estimate	SE	z-value	p
Intercept	-2.636	.099	-26.592	0
Anode Position	-.049	.18	-.272	.789
Verb Type	-.165	.151	-1.095	.274
Resp. Hand	.136	.148	.915	.36
Anode Position x Verb Type	.354	.248	1.427	.154
Anode Position x Resp. Hand	-.34	.252	-1.35	.177
Verb Type x Resp. Hand	-.096	.278	-.344	.731
Anode Position x Verb Type x Resp. Hand	-1.07	.466	-2.293	.022

Supplementary Table 5. Submodel for unimanual verbs: High-error items only

	Estimate	SE	z-value	p
Intercept	-2.659	.124	-21.436	0
Anode Position	.122	.229	.535	.593
Resp. Hand	.038	.215	.178	.859

Anode Position x Resp. Hand	-.804	.347	-2.319	.02

Supplementary Table 6. Submodel for unimanual verbs, left cathode: High-error items only

	Estimate	SE	z-value	p
Intercept	-2.59	.165	-15.688	0
Resp. Hand	-.412	.261	-1.581	.114

Supplementary Table 7. Submodel for unimanual verbs, left anode: High-error items only

	Estimate	SE	z-value	p
Intercept	-2.806	.211	-13.277	0
Resp. Hand	.616	.369	1.667	.095

Supplementary Table 8. Submodel for abstract verbs: High-error items only

	Estimate	SE	z-value	p
Intercept	-2.589	.126	-20.550	0
Anode Position	-.281	.215	-1.306	.192
Resp. Hand	.229	.211	1.082	.279
Anode Position x Resp. Hand	.203	.362	.561	.575

Supplementary Table 9. Full model: Low error items only

	Estimate	SE	z-value	p
Intercept	-3.865	.162	-23.92	0
Anode Position	-.09	.258	-.35	.726
Verb Type	-.216	.269	-.803	.422
Resp. Hand	-.171	.31	-.55	.583
Anode Position x Verb Type	.083	.429	.194	.846
Anode Position x Resp. Hand	.115	.49	.234	.815
Verb Type x Resp. Hand	.156	.523	.299	.765
Anode Position x Verb Type x Resp. Hand	-.192	.737	-.261	.794
